# Extreme thermal stability of the antiGFP nanobody – GFP complex

**DOI:** 10.1186/s13104-023-06382-3

**Published:** 2023-06-20

**Authors:** Balázs Kakasi, Eszter Gácsi, Hajnalka Jankovics, Ferenc Vonderviszt

**Affiliations:** 1 Bio-Nanosystems Laboratory, Research Institute of Biomolecular and Chemical Engineering, Faculty of Engineering, University of Pannonia, Veszprém, Hungary; 2 Institute of Technical Physics and Materials Science, Centre for Energy Research, Budapest, Hungary

**Keywords:** Superfolder GFP, GFP enhancer nanobody, Structural stability, CD, Fluorescence

## Abstract

**Objective:**

The green fluorescent protein (GFP) and its derivatives are widely used in biomedical research. The manipulation of GFP-tagged proteins by GFP-specific binders, e.g. single-domain antibodies (nanobodies), is of increasing significance. It is therefore important to better understand the properties of antiGFP-GFP interaction in order to establish methodological applications. In this work the interaction of superfolder GFP (sfGFP) and its enhancer nanobody (aGFP_enh_) was characterized further.

**Results:**

Previous calorimetric experiments demonstrated that the aGFP_enh_ nanobody binds strongly to sfGFP with a nanomolar affinity. Here we show that this interaction results in a substantial structural stabilization of aGFP_enh_ reflected in a significant increase of its melting temperature by almost 30 °C. The thermal stability of the sfGFP-aGFP_enh_ complex is close to 85 °C in the pH range 7.0–8.5. For therapeutic applications thermoresistance is often an essential factor. Our results suggest that methodologies based on GFP-aGFP interaction can be applied under a wide range of physicochemical conditions. The aGFP_enh_ nanobody seems to be suitable for manipulating sfGFP-labeled targets even in extreme thermophilic organisms.

## Introduction

Single domain antibodies, also known as nanobodies (NBs), have numerous applications in research, diagnostics and therapy [1–6]. They are small binding proteins typically comprising of 110–130 residues. They are derived by directed evolution from the VHH antigen binding domain of the unique heavy chain antibodies found in Camelidae. NBs exhibit several superior properties compared to conventional antibody scaffolds, such as small size, bacterial production, enhanced solubility and stability, low immunogenicity. Many NBs were developed to target soluble extracellular or intracellular proteins, and can be used to block or manipulate a variety of biological processes [5]. They can be used to reprogram cells or report on various aspects of cell function [6].

Green fluorescent protein (GFP) from the jelly-fish *Aequorea victoria* and its derivatives are widely used in biomedical research [7, 8]. GFP is composed of 238 amino acids. A key sequence of Ser–Tyr–Gly at positions 65–67 functions as the GFP fluorophore. Correct folding of the GFP β-barrel architecture is a prerequisite for formation of the fluorescent chromophore. Superfolder GFP (sfGFP) was developed for robust folding by directed evolution and contains 11 point mutations with respect to the wild-type protein [8]. GFP and its variants are extensively applied to visualize dynamic biological processes in vivo and in vitro [9, 10].

To manipulate GFP-tagged target proteins GFP-specific nanobodies has an emerging importance as a research tool in cell and developmental biology [11–18]. They can be used for tracing and perturbing GFP-tagged proteins of interest, or for dissecting protein localization, signaling pathways, and even morphogen gradients. Anti-GFP NBs fused with subcellular localization signals can be applied to identify protein-protein interactions with tagged bait, or to mislocalize target GFP-tagged proteins. aGFP-NB derivatives were also designed to initiate targeted degradation or influence activity of cellular proteins tagged with GFP.

The GFP-enhancer nanobody (aGFP_enh_) can bind GFP and its superfolder variant sfGFP with nanomolar affinity, and this interaction leads to a substantial (~ 1.5-fold) fluorescence enhancement [19, 20]. In order to characterize GFP-nanobody interaction in more detail and better understand the structural consequences of the interaction, we investigated the thermal stability of sfGFP-aGFP_enh_ complex. Our observation demonstrates that the structural stability of the nanobody is greatly enhanced by binding to GFP.

## Materials and methods

### Sample preparation

C-terminally His-tagged sfGFP was produced as described earlier [21]. Briefly, recombinant sfGFP-His6 was overexpressed in *E. coli* BL21-Gold (DE3) cells, then purified by Ni-affinity chromatography.

Nanobody cloning, overexpression and purification was done in a similar way as described in Reider et al. (2021) [22]. The coding sequence of the anti-GFP enhancer nanobody [20] was codon optimized for *E. coli*, the gene was synthetized by Genscript (Piscataway, New Jersey, United States) and cloned into a pET23b expression vector. The aGFP_enh_ protein was produced in Shuffle T7 Express *E. coli* (New England Biolabs, Ipswich, Massachusetts, US) cells and purified by immobilized metal affinity chromatography on a 5 ml HiTrap Chelating column (GE Healthcare, Chicago, Illinois, US).

Protein concentrations of aGFP_enh_ samples were determined by absorption measurement at 280 nm using a molar extinction coefficient of 2.75 × 10^4^ M^− 1^ cm^− 1^ calculated from the amino acid content by the ProtParam program [23]. Protein concentration of sfGFP solutions from the absorbance at 488 nm was calculated using the extinction coefficient of ε_488_ = 5.6 × 10^4^ M^− 1^ cm^− 1^ [21]. Protein samples were prepared in the following buffers: 10 mM citrate-phosphate buffer (pH 5.5), 10 mM phosphate buffer (pH 7.0), and 10 mM Tris-HCl buffer (pH 8.5). The samples used for the measurements contained the same molar concentration (0.0074 mM) of each tested protein component. Purity of protein samples was checked by SDS-PAGE using Coomassie blue R-250 staining.

### CD spectroscopy

Heat stability curves were recorded by a Jasco (Tokyo, Japan) J-1100 spectropolarimeter using a programmable thermoregulated (Peltier PTC-514) cell holder. The measurements were carried out in 0.1 cm pathlength quartz cuvettes over the 20–95 °C temperature range at a fixed wavelength of 204 nm with a heating rate of 1 °C/min. Each sample was measured at different pH values in triplicates.

### Fluorimetry

Fluorescence measurements were performed with a Fluoromax-2 (ISA Jobin-Yvon, Edison, New Jersey, USA) fluorescence spectrophotometer connected to a programmable Julabo (Seelbach, Germany) F25HP heating circulator. Emission spectra were obtained by applying excitation at 488 nm and recording fluorescence intensity in the 500–600 nm wavelength region. In temperature scan experiments the fluorescence intensity was recorded at 508 nm wavelength with 488 nm excitation using 1 °C/min heating rate.

## Results and discussion

Previous studies have shown that anti-GFP_enh_ binds to GFP with a nanomolar affinity [20], resulting in a significant increase in GFP fluorescence [19]. Our own calorimetric and fluorescence spectroscopy measurements confirmed these observations [21]. However, no attempts have been made to investigate the thermal stability of the aGFP_enh_-sfGFP complex.

**Fig. 1:**
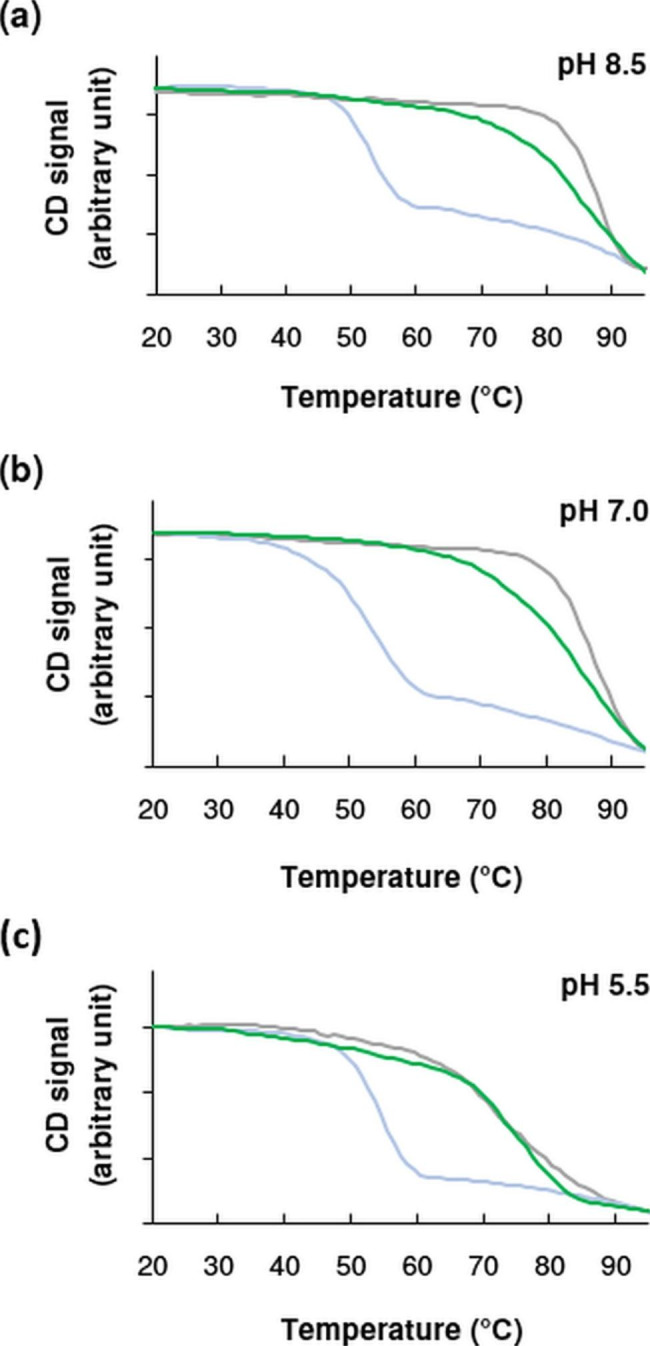
Stability of sfGFP (grey), aGFP_enh_ (blue) and sfGFP-aGFP_enh_ (1:1 molar ratio) (green) samples against thermal denaturation as monitored by far-UV CD spectroscopy at 204 nm. Melting profiles were obtained at pH (**a**) 8.5, (**b**) 7.0 and (**c**) 5.5 with a heating rate of 1 °C /min. For clarity, the start of each melting curve has been shifted to the same point and their magnification has been adjusted so that the end points also coincide

Folding enhanced variants of GFP (eGFP, sfGFP) are highly stable proteins with a β-barrel structure that unfolds above 80 °C under physiological conditions [24]. According to our CD temperature scan measurements (Fig. 1), the apparent denaturation temperature of sfGFP is 86.1 °C (pH 7.0) determined from the inflection point of the melting curve (Table 1). Increasing the pH to 8.5 has no significant effect on the structural stability of sfGFP, while the molecule destabilizes slightly at pH 5.5 and unfolds around 74 °C (Fig. 1c).

**Table 1:** Apparent melting temperatures of sfGFP, aGFP_enh_ and sfGFP-aGFP_enh_ (1:1 molar ratio) samples defined by the inflection point of CD temperature scans

Sample	pH 5.5	pH 7.0	pH 8.5
aGFP_enh_	54.5 °C	52.2 °C	52.5 °C
sfGFP	73.6 °C	86.1 °C	86.0 °C
sfGFP-aGFP_enh_	74.8 °C	84.8 °C	85.1 °C

Nanobodies are reported to show varying stabilities typically in the 45–70 °C range [25]. As compared to sfGFP, thermal stability of aGFP_enh_ is significantly lower and it melts around 53 °C. When tested at different pH values (pH 8.5, pH 5.5), we found that the stability of aGFP_enh_ does not change significantly over the pH range of 5.5–8.5 (Table 1).

Examining the CD melting curve of the 1:1 (molar ratio) aGFP_enh_-sfGFP mixture, we see that the unfolding step of isolated aGFP_enh_ disappears below 60 °C and the complex loses its ordered structure at much higher temperatures close to the denaturation range of sfGFP. In the denaturation region, the slope of the melting curve is less steep compared to that of sfGFP, suggesting that the binding of aGFP_enh_ has a small destabilizing effect on the sfGFP structure, slightly reducing its structural cooperativity. Table 1 summarizes the denaturation temperatures for aGFP_enh_, sfGFP and the aGFP_enh_-sfGFP complex at the pH values tested. These results clearly show that the structure of aGFP_enh_ is significantly stabilized by the interaction with the sfGFP protein, and the complex melts well above 80 °C in the pH range of 7.0–8.5.

**Fig. 2:**
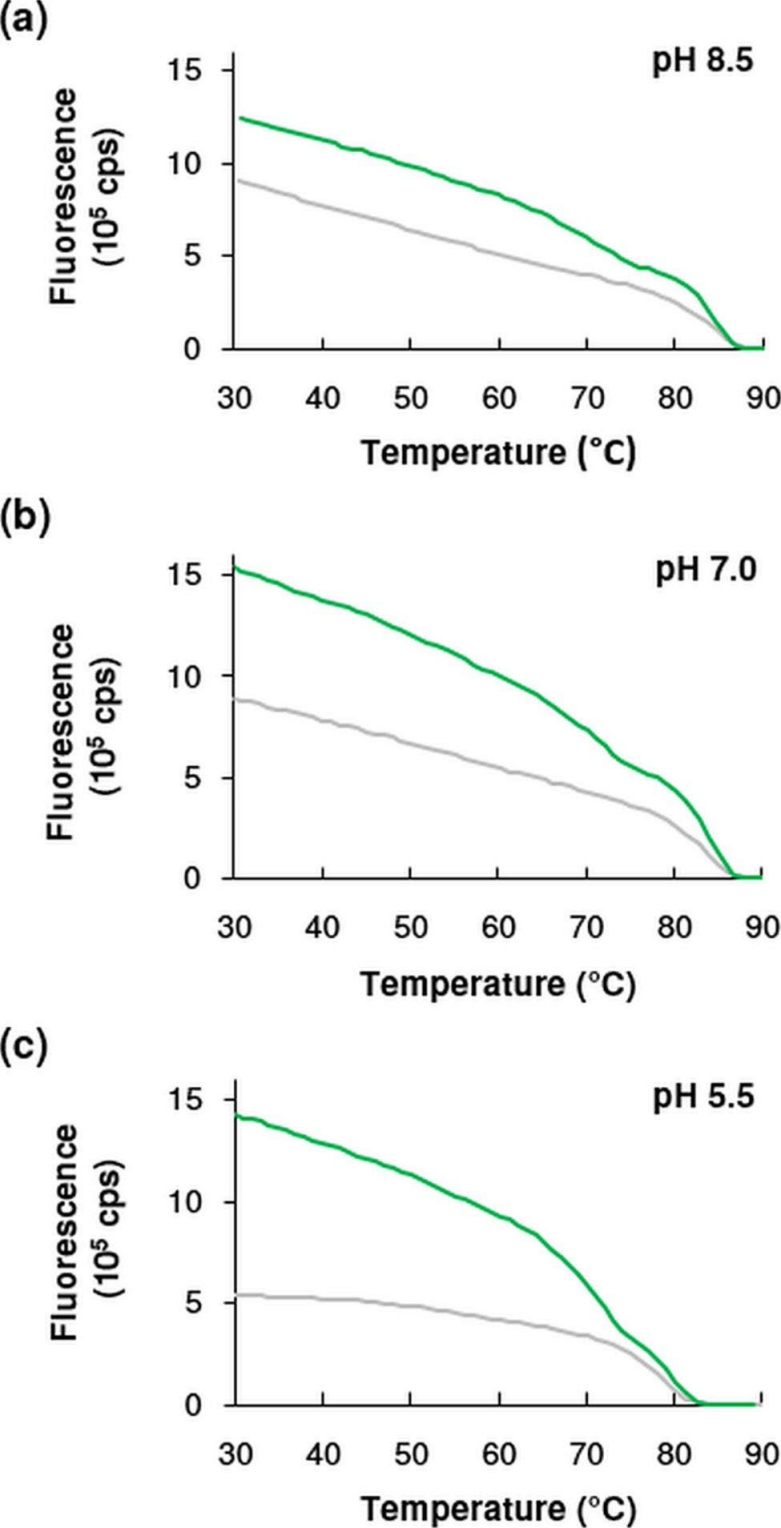
Thermal denaturation of sfGFP in the presence (green) and absence (grey) of aGFP_enh_ at pH (**a**) 8.5, (**b**) 7.0 and (**c**) 5.5 as followed by intrinsic fluorescence intensity measurement at 508 nm. Scanning rate was 1 °C/min. Measurements were done at the same molar concentration (0.0074 mM) for both components

Fluorescence spectroscopy measurements also confirm the high thermal stability of the sfGFP - aGFP_enh_ complex. The fluorescence activity of sfGFP is very similar at pH 7.0 and 8.5, while it decreases significantly by almost 40% at pH 5.5 (Fig. 2), which may be related to a change in structural stability. Indeed, at this pH our CD melting curves also suggested some structural destabilization reflected in a decreased melting temperature.

The binding of aGFP_enh_ to sfGFP is known to result in a substantial increase in fluorescence intensity measured at 508 nm [19]. We found that the degree of enhancement is highly pH dependent: while binding of aGFP_enh_ increased GFP fluorescence at 30 °C by 2.6 times at pH 5.5, this multiplication factor decreased to 1.7 and 1.4 at pH 7.0 and pH 8.5, respectively (Fig. 2). The increased fluorescence intensity of sfGFP in the presence of aGFP_enh_ over a wide range of temperature clearly shows the existence of complex formation. The fluorescence melting curves of the sfGFP-aGFP_enh_ complex show a 2-phase behavior at all three pHs tested. We have no explanation for this observation. Above 70 °C, the enhancing effect of aGFP_enh_ binding is significantly weakened, but still clearly persists. As demonstrated by Fig. 2, the increased fluorescence intensity is maintained until the sfGFP is fully unfolded and loses its fluorescence. This shows that aGFP_enh_ is bound to its partner throughout and they cooperatively lose their ordered structure at elevated temperatures.

In conclusion, our results show that aGFP_enh_ and sfGFP form a stable complex over a wide pH and temperature range. The interaction greatly stabilizes the structure of aGFP_enh_. Upon heating, the two partners cooperatively lose their ordered structure well above 70 °C, close to the melting point of isolated sfGFP. Thus, the aGFP_enh_ nanobody seems to be suitable for manipulating sfGFP-labeled targets even in thermophilic organisms.

### Limitations

In this work, thermal stability of the complex of GFP enhancer nanobody and the superfolder GFP was investigated. The results obtained are not necessarily applicable for the interaction of other GFP variants with different GFP-specific single-domain antibodies.

We observed that the structure of the aGFP_enh_ nanobody is significantly stabilized by the interaction with the sfGFP protein and found that the fluorescence enhancement caused by aGFP_enh_ binding to sfGFP is highly pH dependent, but the structural background of these effects has not been investigated.

## Data Availability

The datasets used and analyzed during the current study are available from the corresponding author on reasonable request.

## Abbreviations

aGFP_enh_: Enhancer anti-GFP nanobody
CD: Circular dichroism.
NB: Nanobody, single domain antibody
sfGFP: Superfolder Green Fluorescent Protein
